# 9-(Dicyano­methyl­idene)fluorene–tetra­thia­fulvalene (1/1)

**DOI:** 10.1107/S1600536812008124

**Published:** 2012-03-03

**Authors:** Amparo Salmerón-Valverde, Sylvain Bernès

**Affiliations:** a Centro de Química del Instituto de Ciencias, Benemérita Universidad Autónoma de Puebla, Ciudad Universitaria, San Manuel, 72570 Puebla, Pue., Mexico; b DEP Facultad de Ciencias Químicas, UANL, Guerrero y Progreso S/N, Col. Treviño, 64570 Monterrey, N.L., Mexico

## Abstract

The title compound, C_16_H_8_N_2_·C_6_H_4_S_4_, crystallizes with the fluorene derivative placed in a general position and two half tetra­thia­fulvalene (TTF) mol­ecules, each completed to a whole mol­ecule through an inversion center. The fluorene ring system is virtually planar (r.m.s. deviation from the mean plane = 0.027 Å) and the dicyano group is twisted from the fluorene plane by only 3.85 (12)°. The TTF mol­ecules are also planar, and their central C=C bond lengths [1.351 (8) and 1.324 (7) Å] compare well with the same bond length in neutral TTF (*ca* 1.35 Å). These features indicate that no charge transfer occurs between mol­ecules in the crystal; the compound should thus be considered a cocrystal rather than an organic complex. This is confirmed by the crystal structure, in which no significant stacking inter­actions are observed between mol­ecules.

## Related literature

For organic conductors based on TTF and a π^*^-acceptor mol­ecule, see: Saito & Ferraris (1980 ▶); Wright (1995 ▶). For structures of dicyano­fulvenes, see: Andrew *et al.* (2010 ▶). For the accurate structure of TTF, see: Batsanov (2006 ▶). For charge-transfer complexes related to the title cocrystal, see: Salmerón-Valverde *et al.* (2003 ▶); Salmerón-Valverde (2008 ▶).
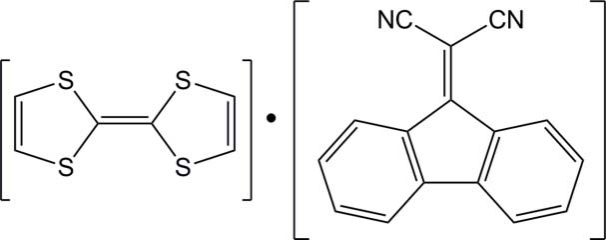


## Experimental

### 

#### Crystal data

C_16_H_8_N_2_·C_6_H_4_S_4_
*M* *_r_* = 432.58
Triclinic, 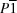

*a* = 7.9919 (11) Å
*b* = 9.3696 (14) Å
*c* = 14.195 (2) Å
α = 94.525 (12)°
β = 103.687 (12)°
γ = 103.252 (12)°
*V* = 995.3 (2) Å^3^
*Z* = 2
Mo *K*α radiation
μ = 0.49 mm^−1^
*T* = 296 K
0.22 × 0.20 × 0.03 mm

#### Data collection

Bruker P4 diffractometer
Absorption correction: ψ scan (*XSCANS*; Siemens, 1996 ▶) *T*_min_ = 0.650, *T*_max_ = 0.688
5766 measured reflections
3493 independent reflections
1541 reflections with *I* > 2σ(*I*)
*R*_int_ = 0.062
2 standard reflections every 48 reflections intensity decay: 14%

#### Refinement

*R*[*F* ^2^ > 2σ(*F* ^2^)] = 0.048
*wR*(*F* ^2^) = 0.119
*S* = 0.95
3493 reflections
254 parameters
H-atom parameters constrained
Δρ_max_ = 0.21 e Å^−3^
Δρ_min_ = −0.21 e Å^−3^

### 

Data collection: *XSCANS* (Siemens, 1996 ▶); cell refinement: *XSCANS*; data reduction: *XSCANS*; program(s) used to solve structure: *SHELXTL-Plus* (Sheldrick, 2008 ▶); program(s) used to refine structure: *SHELXTL-Plus*; molecular graphics: *SHELXTL-Plus* and *Mercury* (Macrae *et al.*, 2008 ▶); software used to prepare material for publication: *SHELXTL-Plus*.

## Supplementary Material

Crystal structure: contains datablock(s) I, global. DOI: 10.1107/S1600536812008124/qm2055sup1.cif

Structure factors: contains datablock(s) I. DOI: 10.1107/S1600536812008124/qm2055Isup2.hkl

Supplementary material file. DOI: 10.1107/S1600536812008124/qm2055Isup3.mol

Supplementary material file. DOI: 10.1107/S1600536812008124/qm2055Isup4.cml

Additional supplementary materials:  crystallographic information; 3D view; checkCIF report

## Figures and Tables

**Fig. 1.**
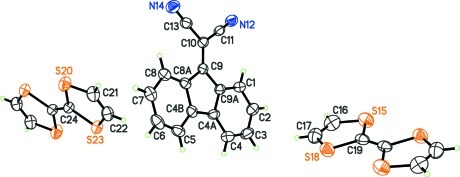
Molecular structure of the title compound, with displacement ellipsoids at the 30% probability level. Unlabelled atoms are generated through inversion centers.

**Fig. 2.**
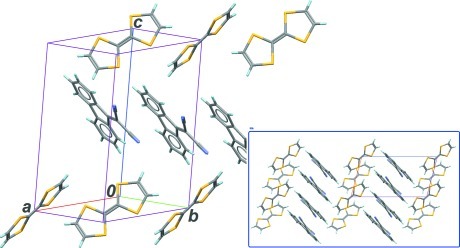
The crystal structure of the title compound, viewed in two orientations. The inset shows two layers of DCF molecules sandwiched by three layers of TTF molecules.

## supplementary crystallographic information

### Comment

There is a vast literature dealing with the organic charge-transfer complexes based on the emblematic π-donor tetrathiafulvalene (TTF) and TTF derivatives. Generally, research in this field is carried out with the hope of obtaining organic materials exhibiting metallic conductivity. It is now known that two essential conditions are required for obtaining such conductivity: *i*) partial oxidation and reduction of the donor and acceptor molecules, respectively. The difference between the redox potentials of the molecules should be less than *ca*. 0.34 V (Saito & Ferraris, 1980); *ii*) molecules must be stacked in the solid state, forming one-dimensional or pseudo one-dimensional crystal structures. The mode of stacking and distances separating molecules along a stack must be suitable for charge-transfer (Wright, 1995). The title compound was formed by mixing TTF and a potential π^*^-acceptor molecule derived from fluorene, namely 9-(dicyanomethylene)fluorene (DCF hereafter). The X-ray structure of the resulting compound, TTF.DCF, shows that condition *ii*) is not present in the structure.

The asymmetric unit includes one DCF molecule, placed in a general position, and two half-TTF molecules, each close to an inversion center, generating the TTF.DCF chemical composition (Fig. 1). The DCF moiety is almost planar, with a r.m.s. deviation of 0.027 Å for the mean plane of the fluorene ring (13 C atoms). The dicyanomethylene plane is twisted by 3.85 (12)° from the fluorene ring, and the C═C bond length in this group, 1.352 (5) Å, is similar to those found in other dicyanomethylene derivatives (*e.g*. Andrew *et al.*, 2010). The same is observed for TTF molecules, giving r.m.s. deviations of 0.037 and 0.020 Å for TTF-1 (S15···C19 and symmetry related atoms) and TTF-2 (S20···C24 and symmetry related atoms), respectively. The central C═C bond lengths are 1.351 (8) and 1.324 (7) Å, no longer that the same bond in neutral TTF, *ca*. 1.35 Å (Batsanov, 2006). These features indicate that molecules are not involved in charge-transfer in the solid state. This is fully confirmed with the crystal structure (Fig. 2). TTF and DCF are segregated in different layers parallel to the (001) plane (Fig. 2, inset), the separation between planes being *c*/2 = 7.1 Å. In the TTF layers, molecules are arranged in a herringbone pattern, avoiding π-π interactions. In the DCF layers, two molecules related by inversion are parallel and the separation between mean-planes for each molecule is relatively short, 3.401 Å. However, DCF molecules are slipped along the stack, and the distance between the centroids of two inversion-related DCF is 3.834 (1) Å. Such an arrangement does not favor π-π interactions for this component.

Spectroscopic data (Salmerón-Valverde, 2008) are consistent with the observed crystal structure. In the solid state, the IR vibration of the cyano groups in TTF.DCF is not shifted with respect to the same vibration in pure DCF (2224 cm^-1^), while a significant shift is expected for an actual charge-transfer complex (Salmerón-Valverde *et al.*, 2003). In the same way, the central C═C bond in TTF, which is known to be sensitive to charge-transfer, is also unaffected when the cocrystal TTF.DCF is formed (ν_C__═__C_: 1527 cm^-1^). In solution, no charge-transfer band is observed in the visible region for TTF.DCF, at any dilution in CH_3_CN.

### Experimental

Solutions of DCF (7.8 mg, 0.034 mmol) in hot CH_3_CN (2.5 ml) and TTF (7 mg, 0.034 mmol) in CH_3_CN (1.8 ml) were mixed and transferred in a test tube (12 × 1.5 cm). Solvent was slowly evaporated in the dark, over 10 days. After all solvent had evaporated, most of the crystals collected on the wall of the test tube were starting components, which present characteristic colors: yellow for TTF and orange for DCF. However, few green crystals of TTF.DCF were produced, with an approximate yield of 25%.

### Refinement

All H atoms were placed in idealized positions and refined as riding to their carrier C atoms, with C—H bond lengths fixed to 0.93 Å. Isotropic displacement parameters for H atoms were calculated as *U*_iso_(H) = 1.2*U*_eq_(carrier C atom).

### Crystal data

**Table:**

C_16_H_8_N_2_·C_6_H_4_S_4_	*Z* = 2
*M**_r_* = 432.58	*F*(000) = 444
Triclinic, *P*1	*D*_x_ = 1.443 Mg m^−^^3^
Hall symbol: -P 1	Melting point: 403 K
*a* = 7.9919 (11) Å	Mo *K*α radiation, λ = 0.71073 Å
*b* = 9.3696 (14) Å	Cell parameters from 57 reflections
*c* = 14.195 (2) Å	θ = 4.0–12.2°
α = 94.525 (12)°	µ = 0.49 mm^−^^1^
β = 103.687 (12)°	*T* = 296 K
γ = 103.252 (12)°	Plate, green
*V* = 995.3 (2) Å^3^	0.22 × 0.20 × 0.03 mm

### Data collection

**Table:**

Bruker P4 diffractometer	1541 reflections with *I* > 2σ(*I*)
Radiation source: X-ray	*R*_int_ = 0.062
Graphite monochromator	θ_max_ = 25.0°, θ_min_ = 2.3°
2θ/ω scans	*h* = −9→3
Absorption correction: ψ scan (*XSCANS*; Siemens, 1996)	*k* = −10→10
*T*_min_ = 0.650, *T*_max_ = 0.688	*l* = −16→16
5766 measured reflections	2 standard reflections every 48 reflections
3493 independent reflections	intensity decay: 14%

### Refinement

**Table:**

Refinement on *F*^2^	Secondary atom site location: difference Fourier map
Least-squares matrix: full	Hydrogen site location: inferred from neighbouring sites
*R*[*F*^2^ > 2σ(*F*^2^)] = 0.048	H-atom parameters constrained
*wR*(*F*^2^) = 0.119	*w* = 1/[σ^2^(*F*_o_^2^) + (0.0414*P*)^2^] where *P* = (*F*_o_^2^ + 2*F*_c_^2^)/3
*S* = 0.95	(Δ/σ)_max_ < 0.001
3493 reflections	Δρ_max_ = 0.21 e Å^−^^3^
254 parameters	Δρ_min_ = −0.21 e Å^−^^3^
0 restraints	Extinction correction: *SHELXTL-Plus* (Sheldrick, 2008), Fc^*^=kFc[1+0.001xFc^2^λ^3^/sin(2θ)]^-1/4^
0 constraints	Extinction coefficient: 0.0125 (17)
Primary atom site location: structure-invariant direct methods	

### Fractional atomic coordinates and isotropic or equivalent isotropic displacement parameters (Å^2^)

**Table:**

	*x*	*y*	*z*	*U*_iso_*/*U*_eq_	
C1	0.0179 (5)	0.9147 (5)	0.3451 (3)	0.0587 (11)	
H1A	−0.0058	0.9988	0.3201	0.070*	
C2	−0.0704 (6)	0.7737 (5)	0.2941 (3)	0.0718 (14)	
H2A	−0.1543	0.7635	0.2345	0.086*	
C3	−0.0341 (7)	0.6488 (5)	0.3318 (4)	0.0767 (14)	
H3A	−0.0935	0.5557	0.2967	0.092*	
C4	0.0888 (6)	0.6601 (5)	0.4206 (4)	0.0694 (13)	
H4A	0.1116	0.5755	0.4452	0.083*	
C4A	0.1776 (5)	0.7992 (5)	0.4723 (3)	0.0538 (11)	
C4B	0.3151 (5)	0.8434 (5)	0.5651 (3)	0.0521 (11)	
C5	0.3937 (6)	0.7596 (5)	0.6298 (4)	0.0643 (12)	
H5A	0.3570	0.6567	0.6188	0.077*	
C6	0.5283 (6)	0.8334 (6)	0.7112 (4)	0.0721 (14)	
H6A	0.5844	0.7783	0.7541	0.087*	
C7	0.5818 (6)	0.9857 (6)	0.7309 (3)	0.0676 (13)	
H7A	0.6712	1.0319	0.7869	0.081*	
C8	0.5017 (5)	1.0703 (5)	0.6668 (3)	0.0589 (11)	
H8A	0.5372	1.1732	0.6799	0.071*	
C8A	0.3686 (5)	1.0006 (5)	0.5833 (3)	0.0495 (10)	
C9	0.2619 (5)	1.0585 (5)	0.5019 (3)	0.0448 (10)	
C9A	0.1426 (5)	0.9265 (4)	0.4344 (3)	0.0485 (10)	
C10	0.2732 (5)	1.2023 (5)	0.4915 (3)	0.0489 (10)	
C11	0.1603 (6)	1.2507 (4)	0.4121 (3)	0.0545 (11)	
N12	0.0739 (5)	1.2952 (4)	0.3509 (3)	0.0719 (11)	
C13	0.3997 (6)	1.3228 (5)	0.5590 (3)	0.0577 (12)	
N14	0.5000 (5)	1.4216 (4)	0.6109 (3)	0.0782 (12)	
S15	0.28023 (18)	0.60731 (16)	−0.03391 (10)	0.0884 (5)	
C16	0.1970 (7)	0.6242 (6)	0.0666 (4)	0.0943 (17)	
H16A	0.0993	0.6633	0.0638	0.113*	
C17	0.2751 (7)	0.5805 (6)	0.1473 (4)	0.0866 (16)	
H17A	0.2330	0.5867	0.2028	0.104*	
S18	0.45887 (18)	0.51072 (15)	0.14801 (9)	0.0832 (5)	
C19	0.4457 (5)	0.5248 (5)	0.0235 (3)	0.0636 (13)	
S20	0.26100 (15)	1.13111 (14)	0.99343 (9)	0.0784 (4)	
C21	0.3288 (6)	1.0451 (6)	0.9025 (3)	0.0742 (14)	
H21A	0.4389	1.0845	0.8909	0.089*	
C22	0.2207 (6)	0.9222 (5)	0.8498 (3)	0.0693 (13)	
H22A	0.2525	0.8725	0.8001	0.083*	
S23	0.01752 (15)	0.85519 (14)	0.87520 (8)	0.0661 (4)	
C24	0.0573 (5)	0.9972 (4)	0.9734 (3)	0.0517 (11)	

### Atomic displacement parameters (Å^2^)

**Table:**

	*U*^11^	*U*^22^	*U*^33^	*U*^12^	*U*^13^	*U*^23^
C1	0.062 (3)	0.048 (3)	0.066 (3)	0.012 (2)	0.016 (3)	0.008 (2)
C2	0.072 (3)	0.063 (3)	0.068 (3)	0.007 (3)	0.010 (3)	−0.002 (3)
C3	0.086 (4)	0.050 (3)	0.086 (4)	0.004 (3)	0.024 (3)	−0.007 (3)
C4	0.081 (3)	0.051 (3)	0.078 (4)	0.020 (3)	0.022 (3)	0.012 (3)
C4A	0.060 (3)	0.041 (3)	0.068 (3)	0.017 (2)	0.028 (2)	0.008 (2)
C4B	0.050 (3)	0.060 (3)	0.054 (3)	0.018 (2)	0.024 (2)	0.013 (2)
C5	0.072 (3)	0.061 (3)	0.076 (3)	0.031 (3)	0.031 (3)	0.028 (3)
C6	0.068 (3)	0.098 (4)	0.073 (4)	0.042 (3)	0.032 (3)	0.040 (3)
C7	0.062 (3)	0.088 (4)	0.058 (3)	0.025 (3)	0.018 (2)	0.023 (3)
C8	0.057 (3)	0.061 (3)	0.063 (3)	0.017 (2)	0.019 (2)	0.016 (3)
C8A	0.048 (2)	0.054 (3)	0.056 (3)	0.018 (2)	0.022 (2)	0.016 (2)
C9	0.045 (2)	0.048 (3)	0.049 (2)	0.017 (2)	0.020 (2)	0.007 (2)
C9A	0.049 (2)	0.047 (3)	0.053 (3)	0.014 (2)	0.020 (2)	0.006 (2)
C10	0.047 (3)	0.049 (3)	0.049 (3)	0.012 (2)	0.009 (2)	0.004 (2)
C11	0.064 (3)	0.041 (3)	0.059 (3)	0.012 (2)	0.022 (3)	−0.002 (2)
N12	0.087 (3)	0.057 (3)	0.066 (3)	0.023 (2)	0.005 (2)	0.004 (2)
C13	0.065 (3)	0.051 (3)	0.062 (3)	0.018 (3)	0.020 (3)	0.015 (2)
N14	0.080 (3)	0.064 (3)	0.077 (3)	0.011 (2)	0.005 (2)	0.002 (2)
S15	0.0775 (9)	0.0981 (11)	0.0898 (10)	0.0303 (8)	0.0098 (8)	0.0282 (8)
C16	0.072 (4)	0.096 (4)	0.103 (4)	0.022 (3)	0.005 (3)	0.002 (4)
C17	0.075 (4)	0.089 (4)	0.086 (4)	0.004 (3)	0.022 (3)	−0.005 (3)
S18	0.0863 (10)	0.0853 (10)	0.0709 (9)	0.0159 (8)	0.0105 (7)	0.0185 (7)
C19	0.064 (3)	0.049 (3)	0.063 (3)	0.000 (2)	0.000 (2)	0.015 (2)
S20	0.0612 (8)	0.0889 (10)	0.0702 (9)	−0.0083 (7)	0.0218 (7)	−0.0093 (7)
C21	0.052 (3)	0.101 (4)	0.069 (3)	0.012 (3)	0.022 (3)	0.012 (3)
C22	0.061 (3)	0.091 (4)	0.065 (3)	0.026 (3)	0.029 (3)	0.013 (3)
S23	0.0628 (8)	0.0728 (9)	0.0583 (7)	0.0136 (6)	0.0153 (6)	−0.0042 (6)
C24	0.049 (3)	0.052 (3)	0.052 (3)	0.012 (2)	0.0123 (19)	0.002 (2)

### Geometric parameters (Å, º)

**Table:**

C1—C9A	1.394 (5)	C9—C10	1.352 (5)
C1—C2	1.395 (5)	C9—C9A	1.482 (5)
C1—H1A	0.9300	C10—C13	1.436 (6)
C2—C3	1.386 (6)	C10—C11	1.442 (6)
C2—H2A	0.9300	C11—N12	1.144 (5)
C3—C4	1.382 (6)	C13—N14	1.147 (5)
C3—H3A	0.9300	S15—C16	1.722 (6)
C4—C4A	1.386 (5)	S15—C19	1.752 (4)
C4—H4A	0.9300	C16—C17	1.312 (6)
C4A—C9A	1.404 (5)	C16—H16A	0.9300
C4A—C4B	1.460 (6)	C17—S18	1.737 (5)
C4B—C5	1.386 (5)	C17—H17A	0.9300
C4B—C8A	1.421 (5)	S18—C19	1.762 (4)
C5—C6	1.384 (6)	C19—C19^i^	1.351 (8)
C5—H5A	0.9300	S20—C21	1.726 (5)
C6—C7	1.378 (6)	S20—C24	1.759 (4)
C6—H6A	0.9300	C21—C22	1.317 (6)
C7—C8	1.392 (5)	C21—H21A	0.9300
C7—H7A	0.9300	C22—S23	1.734 (4)
C8—C8A	1.388 (5)	C22—H22A	0.9300
C8—H8A	0.9300	S23—C24	1.766 (4)
C8A—C9	1.483 (5)	C24—C24^ii^	1.324 (7)
			
C9A—C1—C2	118.5 (4)	C10—C9—C9A	127.4 (4)
C9A—C1—H1A	120.8	C10—C9—C8A	126.8 (4)
C2—C1—H1A	120.8	C9A—C9—C8A	105.8 (3)
C3—C2—C1	120.4 (4)	C1—C9A—C4A	120.6 (4)
C3—C2—H2A	119.8	C1—C9A—C9	130.8 (4)
C1—C2—H2A	119.8	C4A—C9A—C9	108.5 (4)
C4—C3—C2	121.2 (4)	C9—C10—C13	123.1 (4)
C4—C3—H3A	119.4	C9—C10—C11	123.8 (4)
C2—C3—H3A	119.4	C13—C10—C11	113.1 (4)
C3—C4—C4A	119.1 (4)	N12—C11—C10	177.1 (5)
C3—C4—H4A	120.4	N14—C13—C10	178.0 (5)
C4A—C4—H4A	120.4	C16—S15—C19	94.7 (2)
C4—C4A—C9A	120.1 (4)	C17—C16—S15	118.3 (5)
C4—C4A—C4B	130.7 (4)	C17—C16—H16A	120.9
C9A—C4A—C4B	109.2 (4)	S15—C16—H16A	120.9
C5—C4B—C8A	120.8 (4)	C16—C17—S18	118.5 (5)
C5—C4B—C4A	131.0 (4)	C16—C17—H17A	120.8
C8A—C4B—C4A	108.2 (4)	S18—C17—H17A	120.8
C6—C5—C4B	118.1 (4)	C17—S18—C19	93.8 (2)
C6—C5—H5A	120.9	C19^i^—C19—S15	123.0 (5)
C4B—C5—H5A	120.9	C19^i^—C19—S18	122.5 (5)
C7—C6—C5	122.2 (4)	S15—C19—S18	114.6 (2)
C7—C6—H6A	118.9	C21—S20—C24	94.8 (2)
C5—C6—H6A	118.9	C22—C21—S20	118.2 (4)
C6—C7—C8	119.9 (4)	C22—C21—H21A	120.9
C6—C7—H7A	120.1	S20—C21—H21A	120.9
C8—C7—H7A	120.1	C21—C22—S23	118.4 (4)
C8A—C8—C7	119.7 (4)	C21—C22—H22A	120.8
C8A—C8—H8A	120.1	S23—C22—H22A	120.8
C7—C8—H8A	120.1	C22—S23—C24	94.4 (2)
C8—C8A—C4B	119.3 (4)	C24^ii^—C24—S20	123.1 (4)
C8—C8A—C9	132.4 (4)	C24^ii^—C24—S23	122.8 (4)
C4B—C8A—C9	108.3 (4)	S20—C24—S23	114.1 (2)
			
C9A—C1—C2—C3	−0.1 (6)	C4—C4A—C9A—C1	0.4 (6)
C1—C2—C3—C4	0.5 (7)	C4B—C4A—C9A—C1	178.9 (3)
C2—C3—C4—C4A	−0.3 (7)	C4—C4A—C9A—C9	−177.2 (4)
C3—C4—C4A—C9A	−0.1 (6)	C4B—C4A—C9A—C9	1.3 (4)
C3—C4—C4A—C4B	−178.2 (4)	C10—C9—C9A—C1	1.4 (6)
C4—C4A—C4B—C5	−2.0 (7)	C8A—C9—C9A—C1	−178.0 (4)
C9A—C4A—C4B—C5	179.7 (4)	C10—C9—C9A—C4A	178.7 (4)
C4—C4A—C4B—C8A	176.8 (4)	C8A—C9—C9A—C4A	−0.7 (4)
C9A—C4A—C4B—C8A	−1.4 (4)	C9A—C9—C10—C13	−176.9 (4)
C8A—C4B—C5—C6	−1.5 (6)	C8A—C9—C10—C13	2.3 (6)
C4A—C4B—C5—C6	177.3 (4)	C9A—C9—C10—C11	3.1 (6)
C4B—C5—C6—C7	1.9 (6)	C8A—C9—C10—C11	−177.6 (3)
C5—C6—C7—C8	−1.1 (6)	C19—S15—C16—C17	1.9 (5)
C6—C7—C8—C8A	−0.2 (6)	S15—C16—C17—S18	0.8 (6)
C7—C8—C8A—C4B	0.6 (5)	C16—C17—S18—C19	−3.0 (5)
C7—C8—C8A—C9	−179.0 (4)	C16—S15—C19—C19^i^	175.8 (5)
C5—C4B—C8A—C8	0.3 (5)	C16—S15—C19—S18	−3.9 (3)
C4A—C4B—C8A—C8	−178.7 (3)	C17—S18—C19—C19^i^	−175.6 (5)
C5—C4B—C8A—C9	179.9 (3)	C17—S18—C19—S15	4.2 (3)
C4A—C4B—C8A—C9	0.9 (4)	C24—S20—C21—C22	1.5 (4)
C8—C8A—C9—C10	0.1 (6)	S20—C21—C22—S23	0.1 (6)
C4B—C8A—C9—C10	−179.5 (4)	C21—C22—S23—C24	−1.5 (4)
C8—C8A—C9—C9A	179.4 (4)	C21—S20—C24—C24^ii^	178.3 (5)
C4B—C8A—C9—C9A	−0.2 (4)	C21—S20—C24—S23	−2.4 (3)
C2—C1—C9A—C4A	−0.3 (6)	C22—S23—C24—C24^ii^	−178.3 (5)
C2—C1—C9A—C9	176.7 (4)	C22—S23—C24—S20	2.4 (3)

Symmetry codes: (i) −*x*+1, −*y*+1, −*z*; (ii) −*x*, −*y*+2, −*z*+2.
